# Streptococcal inhibitor of complement (SIC) modulates fibrinolysis and enhances bacterial survival within fibrin clots

**DOI:** 10.1074/jbc.RA118.001988

**Published:** 2018-07-12

**Authors:** Inga-Maria Frick, Oonagh Shannon, Ariane Neumann, Christofer Karlsson, Mats Wikström, Lars Björck

**Affiliations:** From the ‡ Department of Clinical Sciences, Lund, Division of Infection Medicine, Lund University, SE-22184 Lund, Sweden and; the § University of Copenhagen, Protein Function and Interactions Group, Novo Nordisk Foundation Center for Protein Research, DK-2200 Copenhagen, Denmark

**Keywords:** thrombin, plasminogen, innate immunity, *Streptococcus pyogenes* (*S. pyogenes*), fibrinolysis, antimicrobial agent, bacterial clearance, group A streptococci, streptococcal inhibitor of complement, virulence

## Abstract

Some strains of the bacterial pathogen *Streptococcus pyogenes* secrete protein SIC (streptococcal inhibitor of complement), including strains of the clinically relevant M1 serotype. SIC neutralizes the effect of a number of antimicrobial proteins/peptides and interferes with the function of the host complement system. Previous studies have shown that some *S. pyogenes* proteins bind and modulate coagulation and fibrinolysis factors, raising the possibility that SIC also may interfere with the activity of these factors. Here we show that SIC interacts with both human thrombin and plasminogen, key components of coagulation and fibrinolysis. We found that during clot formation, SIC binds fibrin through its central region and that SIC inhibits fibrinolysis by interacting with plasminogen. Flow cytometry results indicated that SIC and plasminogen bind simultaneously to *S. pyogenes* bacteria, and fluorescence microscopy revealed co-localization of the two proteins at the bacterial surface. As a consequence, SIC-expressing bacteria entrapped in clots inhibit fibrinolysis, leading to delayed bacterial escape from the clots as compared with mutant bacteria lacking SIC. Moreover, within the clots SIC-expressing bacteria were protected against killing. In an animal model of subcutaneous infection, SIC-expressing bacteria exhibited a delayed systemic spread. These results demonstrate that the bacterial protein SIC interferes with coagulation and fibrinolysis and thereby enhances bacterial survival, a finding that has significant implications for *S. pyogenes* virulence.

## Introduction

The coagulation system is rapidly activated upon vascular injury, whereby a fibrin clot is generated and blood is loss prevented. This system comprises two pathways, the extrinsic (tissue factor pathway) and the intrinsic pathway (also known as the contact system) (see Refs. 1 and 2). Activation of both pathways leads to the generation of thrombin, which acts upon fibrinogen with the formation of a fibrin network (for a review see, Ref. 2). Coagulation is tightly regulated by anticoagulants, which inhibit coagulation factors, and the fibrinolytic system, which degrades the fibrin clot (3, 4). Plasminogen, the main component of the fibrinolytic system, is converted to active plasmin by tissue-type plasminogen activator (tPA)^3^ or by urokinase-type plasminogen activator (uPA) (5). In addition, bacterial activators, such as streptokinase secreted by groups A, C, and G streptococci, bind and activate plasminogen (see Refs. 6 and 7). Whereas tPA cleaves the molecule at a specific site generating the active two-chain plasmin (5), streptokinase causes activation by forming a complex with plasminogen. This complex formation leads to the exposure of the active center in plasminogen, whereby the molecule is activated without any cleavage. Another plasminogen molecule is then bound, and through proteolysis plasmin is generated (8).

As a first line of defense against infecting microorganisms, the innate immune system, including antimicrobial proteins/peptides and complement, plays an important role (9, 10). The coagulation system is closely linked to inflammation and innate immunity (11, 12), and when an inflammatory response is induced at the site of infection and plasma is released, a clot is formed that entraps the bacteria limiting their spread and invasion (13–16). The demonstration that proteolysis of thrombin (17) and thrombin cleavage of bacteria-bound fibrinogen (18) generates peptides with antibacterial activity further links coagulation to innate immunity.

Thrombin generation and local fibrin deposition has been shown to play a role in host defense against the important human pathogen *Streptococcus pyogenes* (group A streptococci) (19) (also see Ref. 20). In addition, it was demonstrated that histidine-rich glycoprotein (HRG) is important for the immobilization and killing of these bacteria by fibrin clots (21). *S. pyogenes* causes a variety of infections from mild skin and throat-infections to severe conditions such as sepsis and necrotizing fasciitis (22). These bacteria are divided into more than 100 serotypes (23) based on variations in a surface protein, the M protein (for review see Ref. 24). Strains of the M1 serotype have been dominant worldwide during the last decades and are frequently isolated from patients with invasive infections (25, 26). M1 strains secrete protein SIC, initially described as an inhibitor of complement activity (27). However, subsequent studies have shown that SIC binds and inhibits the activity of several antimicrobial proteins and peptides (21, 28–33), and the protein also promotes mucosal colonization (34) and epithelial cell adherence (35). In addition, SIC interacts with high molecular weight kininogen (HK), a component of the pro-inflammatory bradykinin-forming pathway of the contact system (see Ref. 1). This interaction interferes with the binding of HK to endothelial cells, which reduces contact system activation and bradykinin generation (36).

Several studies have shown that surface proteins of *S. pyogenes* bind and modulate factors involved in coagulation and fibrinolysis, such as plasminogen, fibrinogen, and coagulation components (22, 37, 38). This and the evolutionary success of M1 strains, which secrete large amounts of SIC (27), raised the question of whether also SIC could interfere with coagulation and fibrinolysis. The results presented below demonstrate that this is the case, adding new aspects to the function of SIC and its potential role in *S. pyogenes* virulence.

## Results

### SIC binds to thrombin and fibrin clots

**Figure 1.**
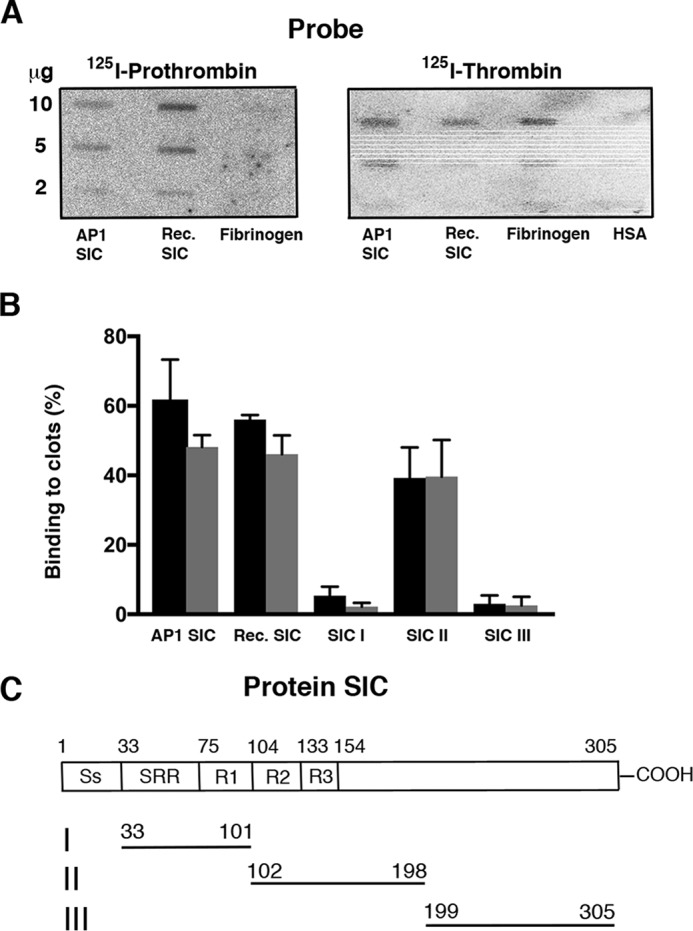
**SIC binds to thrombin and fibrin clots.** *A*, indicated amounts of SIC produced by *S. pyogenes* AP1 bacteria (*AP1 SIC*) or recombinantly expressed (*Rec. SIC*) in *E. coli* and human fibrinogen were applied to PVDF membranes. The membranes were incubated with radiolabeled human prothrombin (*left panel*) or human thrombin (*right panel*), and binding was detected using the Fuji imaging system. HSA was applied as a negative control for binding of thrombin. The experiment with radiolabeled thrombin was performed three times, and a representative image is shown. *B*, clots formed by adding thrombin to fibrinogen solution or human citrate-treated plasma were incubated with intact radiolabeled SIC and SIC fragments I–III. Bound radioactivity is shown as a percentage. *Black columns*, clots formed in fibrinogen solution; *gray columns*, clots formed in plasma. Mean values from three experiments ± S.D. are shown. *C*, schematic structure of SIC showing the signal peptide (*Ss*), which is cleaved off in the mature secreted protein, the short repeat region (*SRR*), and three tandem repeats (*R1–R3*). Shown are fragments I–III of SIC recombinantly expressed in *E. coli*. The choice of localization and size of fragments I–III was based on previous publications (30, 40). The *numbers* refer to amino acid positions in the unprocessed SIC protein containing the signal peptide.

To investigate whether SIC has affinity for thrombin, SIC purified from *S. pyogenes* strain AP1 or produced in *Escherichia coli* using recombinant expression was applied to PVDF filters that were incubated with radiolabeled prothrombin or thrombin. Fibrinogen served as a positive control for thrombin binding and human serum albumin (HSA) as a negative control. As shown in Fig. 1*A*, SIC binds to both prothrombin and thrombin. Using a chromogenic substrate assay, we tested the interference of SIC with the activity of thrombin, but no effect on thrombin activity was observed (data not shown). Next, SIC was incubated with human citrate–treated plasma and the thrombin-induced coagulation time was analyzed. Again, SIC had no effect on the activity of thrombin (data not shown). During clot formation thrombin is incorporated in the clots where it binds to fibrin via nonsubstrate sites in the E and D domains (39). Thus, we tested whether SIC was able to bind to thrombin-induced clots formed both in fibrinogen solution and in human citrate-treated plasma. The two *leftmost sections* of Fig. 1*B* demonstrate that radiolabeled SIC binds to such clots and that the binding of SIC isolated from *S. pyogenes* growth medium or from *E. coli* expressing the *sic* gene shows a similar pattern.

During coagulation and inflammation, neutrophil elastase is released. Previous studies have shown that elastase digestion of SIC results in three major fragments (30, 40). Based on these studies and on the regions of SIC described by Åkesson *et al.* (27), three recombinant SIC fragments were expressed in *E. coli*: SIC I, fragment(33–101); SIC II, fragment(102–198); and SIC III, fragment(199–305). The positions of these fragments are shown in Fig. 1*C*. Using these fragments, the binding of SIC to fibrin clots was mapped to a region covered by fragment II in the central region of the SIC molecule (Fig. 1*B*, *SI–SIII*).

### SIC binds plasminogen and modulates fibrinolysis

**Figure 2.**
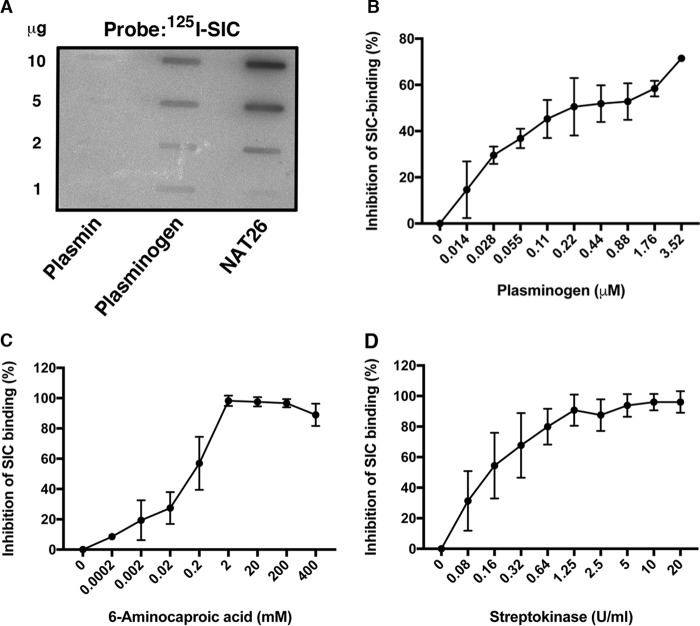
**SIC binds to plasminogen.** *A*, various amounts of plasminogen and plasmin were applied to a PVDF membrane. The membrane was incubated with radiolabeled SIC, and binding was detected using the Fuji imaging system. The kininogen-derived antibacterial peptide NAT26 was used as a positive control. *B–D*, ELISA plates were coated with plasminogen (0.6 μg/ml) overnight. SIC (2 μg/ml) was added together with the indicated concentrations of plasminogen (*B*), 6-aminocaproic acid (*C*), and streptokinase (*D*). Binding of SIC was detected using anti-SIC antibodies followed by peroxidase-conjugated secondary antibodies. Data shown are mean values ± S.D. of at least three experiments.

**Figure 3.**
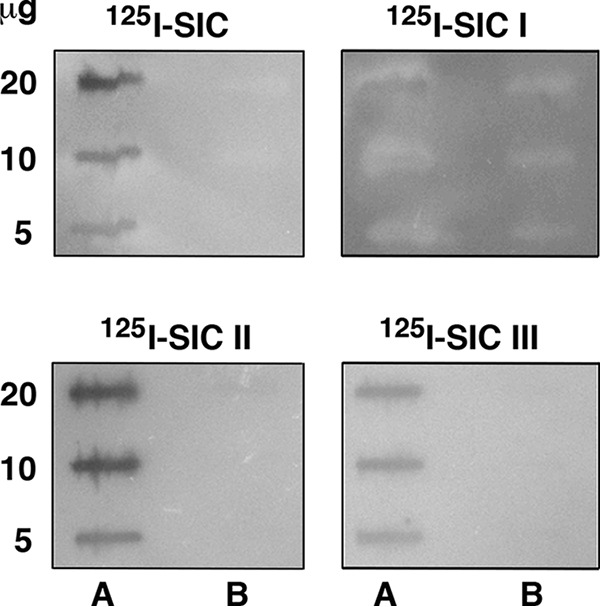
**Mapping of the plasminogen binding site in SIC.** The indicated amounts of plasminogen (*lane A*) and streptokinase (*lane B*) were applied in slots to PVDF membranes. The membranes were incubated with radiolabeled whole SIC and SIC-fragments I, II, and III, respectively. Binding was detected using the Fuji imaging system.

The binding of SIC to fibrin clots (Fig. 1*B*) suggested that SIC might interfere with fibrinolysis. The binding of SIC to plasminogen was thus tested in slot-binding assays, where radiolabeled SIC bound to immobilized plasminogen but not to plasmin, the active form of plasminogen (Fig. 2*A*). The SIC-binding antibacterial peptide NAT26 (41) served as a positive control. SIC also bound to plasminogen immobilized on microtiter plates. This interaction was blocked by plasminogen itself and by 6-aminocaproic acid, suggesting that SIC binds to the lysine-binding sites in the kringle domains of plasminogen (Fig. 2, *B* and *C*). Streptokinase, which forms a complex with plasminogen and thereby causes its activation, also interfered with the binding of SIC to plasminogen (Fig. 2*D*). Moreover, using radiolabeled SIC fragments as probes in the slot-binding assay, the binding site for plasminogen could be mapped to the region of SIC covered by fragments II and III (Fig. 3). No binding to streptokinase was observed (Fig. 3).

**Figure 4.**
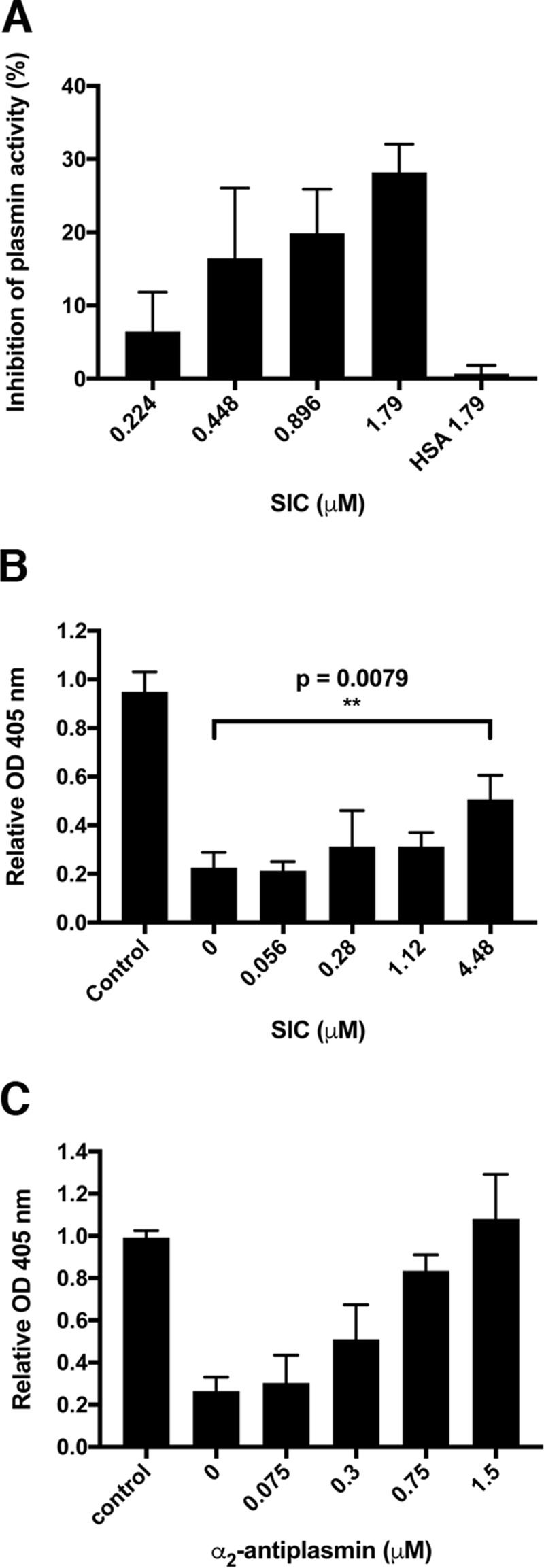
**SIC inhibits fibrinolysis.** *A*, plasminogen (2 μg) was incubated with streptokinase (8 units) and the indicated concentrations of SIC for 1 h at 37 °C. A chromogenic substrate (S-2251) specific for streptokinase-activated plasminogen was added, and incubation continued for another hour at 37 °C. Cleavage of the substrate was analyzed by measuring the absorbance at 405 nm. HSA was used as a negative control. Mean values ± S.D. of three experiments are shown. *B* and *C*, clots were formed by incubating human fibrinogen (150 μg) with human thrombin (1 μg = 3.3 units) in microwell plates at 37 °C for 1.5 h, and the absorbance at 405 nm was measured (buffer control). Plasminogen (final concentration of 2.5 μg/ml) and streptokinase (final concentration of 40 units/ml) were then added together with various concentrations of SIC (*B*) and α_2_-antiplasmin (*C*), and incubation at 37 °C was continued for another 3 h. The absorbance at 405 nm was measured. The absorbance at 0 time point was set to 1 in each individual well, and the absorbance at 3 h was related to the absorbance at 0 time point. Mean values ± S.D. of four experiments are shown. The data in *B* were analyzed statistically using the Mann–Whitney test.

Next, we investigated whether SIC could interfere with the activity of streptokinase-activated plasminogen. First, plasminogen was incubated with streptokinase and SIC at various concentrations, and the cleavage of a chromogenic substrate was determined. As shown in Fig. 4*A*, SIC inhibited the cleavage of the substrate, suggesting that SIC interferes with the activation of plasminogen. In contrast, SIC did not inhibit the activation of plasminogen by tPA (data not shown). To test for whether SIC also modulates fibrinolysis, clots were formed in 96-well plates by incubating fibrinogen with human thrombin. Following clot formation, the optical density was assessed, and then plasminogen and streptokinase were added together with various amounts of SIC. Fig. 4*B* shows that SIC significantly inhibited fibrinolytic activity although not as efficiently as the physiological inhibitor α_2_-antiplasmin (Fig. 4*C*).

### Plasminogen and SIC bind to the surface of S. pyogenes

**Figure 5.**
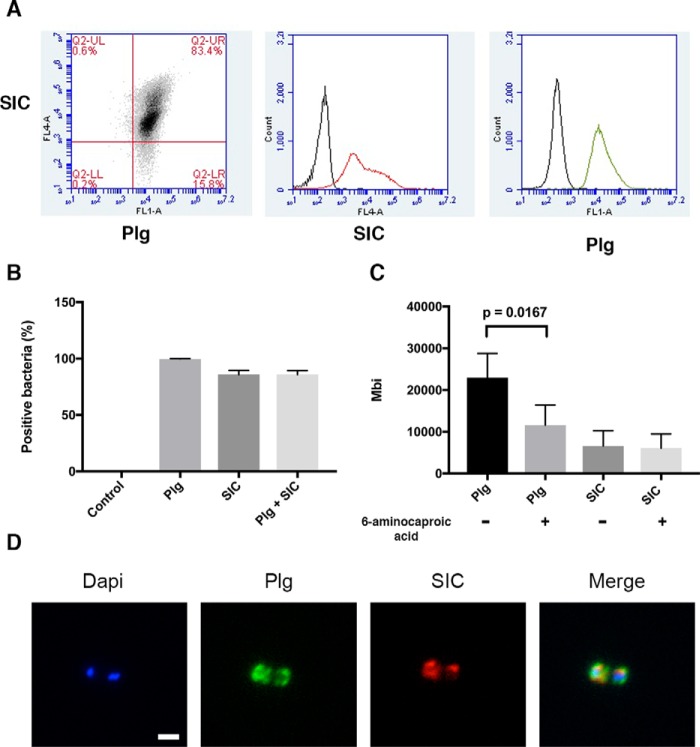
**Plasminogen and SIC bind to *S. pyogenes* bacteria.** *A–C*, stationary phase *S. pyogenes* strain AP1 (2 × 10^7^ cfu/ml in PBS) was incubated with Alexa Fluor 488–plasminogen (*Plg*) and Alexa Fluor 633–SIC for 30 min. Samples were analyzed by flow cytometry. *A*, the *top right quadrant* in the *far left* image represents bacterial cells with associated SIC and plasminogen. FL4-A (*middle*) measures the intensity of SIC binding, and FL1-A (*right*) measures the intensity of plasminogen. For each sample 30,000 events were recorded. *B*, the percentage of bacterial cells positive for plasminogen and/or SIC *C*, the median fluorescence intensity of plasminogen and SIC binding with and without the inhibitor 6-aminocaproic acid. Data shown are mean values ± S.D. of three experiments. The data in *C* were analyzed statistically using the Mann–Whitney test. *D*, *S. pyogenes* AP1 bacterial cells from above were washed with PBS–BSA and adhered onto poly-l-lysine coverslips. Representative fluorescence micrographs are shown. DNA, *blue*; plasminogen, *green*; SIC, *red*; merged image (*Merge*), *yellow* signal indicates co-localization of plasminogen and SIC. Pearson correlation coefficient: 0.844. The *scale bar* represents 2 μm.

Several studies have shown that *S. pyogenes* binds plasminogen via various bacterial surface molecules (see Ref. 37). Moreover, strains of the M1 serotype recruit plasminogen via fibrinogen bound to the M1 protein (42, 43). We used flow cytometry to study the interaction between plasminogen and SIC at the bacterial surface. Here, the AP1 strain was incubated with plasminogen labeled with Alexa Fluor 488, and with protein SIC, labeled with Alexa Fluor 633. Binding to the bacteria was defined as the percentage of bacterial cells that were positive for plasminogen and SIC (Fig. 5*A*). In a parallel experiment we analyzed bacteria that were washed after incubation with the Alexa Fluor–labeled proteins. A similar pattern of binding was obtained (data not shown), and therefore all further experiments were conducted with unwashed bacteria. The number of bacterial cells positive for SIC was slightly lower as compared with bacteria positive for plasminogen (Fig. 5*B*). However, the median fluorescence intensity of plasminogen binding to AP1 bacteria was higher than the median fluorescence intensity of SIC binding (Fig. 5*C*). Furthermore, the addition of 6-aminocaproic acid to bacterial cells significantly decreased the intensity of plasminogen binding but had no effect on the intensity of protein SIC binding (Fig. 5*C*). No decrease in the percentage of bacterial cells positive for plasminogen and SIC binding was observed (data not shown). Experiments were also conducted in the presence of fibrinogen, which did not affect the percentage of bacterial cells positive for plasminogen and SIC binding. However, the median fluorescence intensity was slightly enhanced in the presence of fibrinogen (data not shown). Next, AP1 bacteria incubated with Alexa Fluor–labeled plasminogen and SIC were prepared for fluorescence microscopy. Fig. 5*D* demonstrates that SIC co-localizes with plasminogen at the bacterial surface, with a Pearson correlation coefficient of 0.844 indicating strong positive association.

### SIC delays fibrinolysis and promotes bacterial survival within fibrin clots

**Figure 6.**
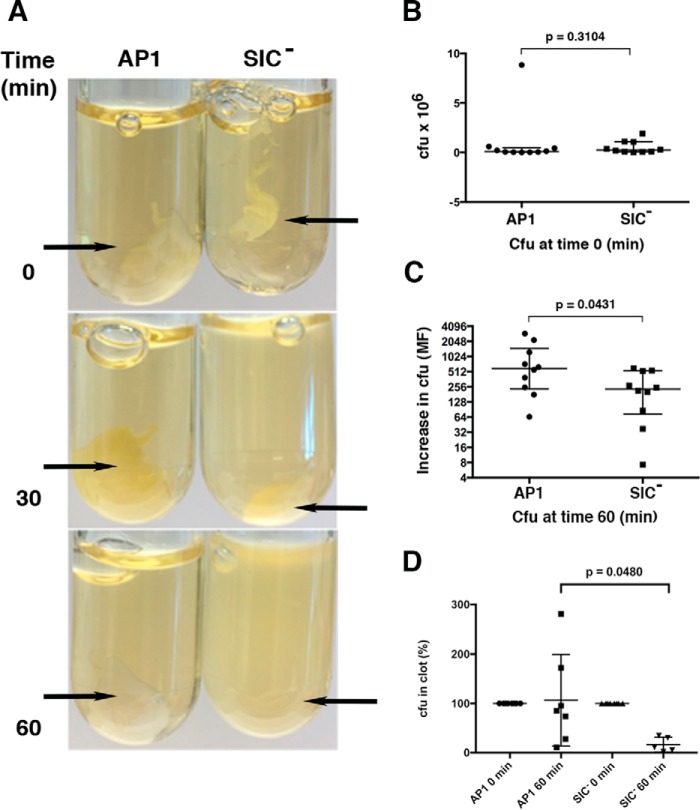
**SIC producing *S. pyogenes* bacteria are retained in clots.** Stationary phase *S. pyogenes* strains AP1 and SIC^−^ (2 × 10^9^ cfu/ml in 13 mm citrate buffer) were incubated with plasma at 37 °C, 5% CO_2_ for 30 min. Human thrombin was added, and incubation continued for another 30 min at 37 °C, 5% CO_2_. The clots formed were washed with citrate buffer, TH broth was added, and the clots were incubated at 37 °C, 5% CO_2_. *A*, at indicated time points the clots were examined visually. *B* and *C*, at time points 0 and 60 min, 10 μl withdrawn from the TH broth surrounding the clots was diluted and spread on TH-agar plates. The plates were incubated overnight, and cfu were counted. The number of cfu between strains in *B* is not statistically significantly different (*p* = 0.3104); the number of cfu between strains in *C* is statistically significantly different (*p* = 0.0431). Data from 10 experiments are shown (median with interquartile range) and were evaluated using the Mann–Whitney test. *D*, clots were formed as described above, and at the indicated time points the clots were washed, the fibrin network was resolved by trypsin treatment, and appropriate dilutions were spread on TH-agar plates. The number of cfu between strains at time point 0 min is not statistically significantly different (*p* > 0.9999); the number of cfu between strains at time point 60 min is statistically significantly different (*p* = 0.0480). Data from eight experiments are shown (median with interquartile range) and were evaluated using the Mann–Whitney test.

**Figure 7.**
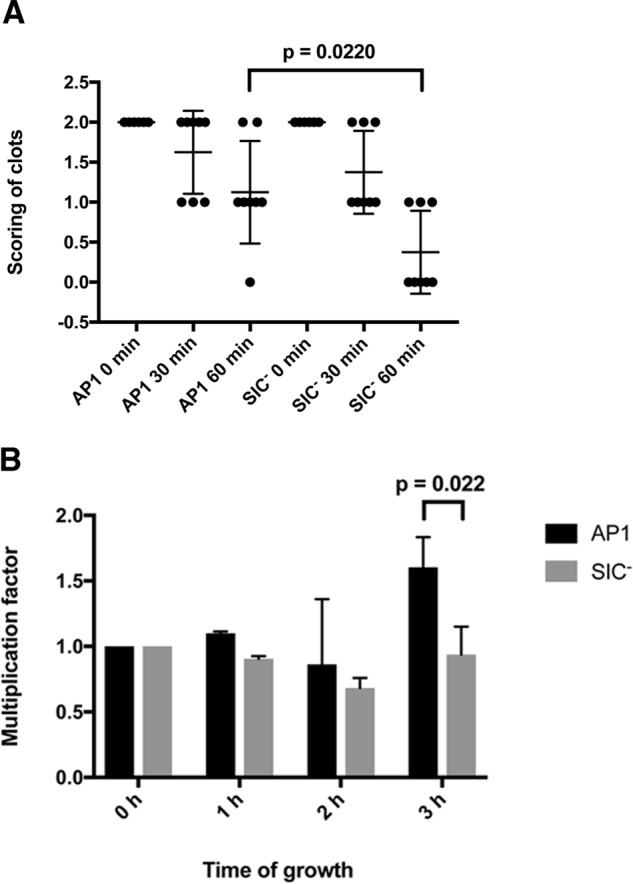
***S. pyogenes* and disintegration of plasma clots and growth in plasma without thrombin.** *A*, stationary phase *S. pyogenes* AP1 bacteria and SIC^−^ bacteria (2 × 10^9^ cfu/ml in 13 mm citrate buffer) were incubated with plasma at 37 °C, 5% CO_2_ for 30 min. Human thrombin was added, and incubation continued for another 30 min at 37 °C, 5% CO_2_. The clots formed were washed with citrate buffer, TH broth was added, and the clots were incubated at 37 °C, 5% CO_2_ (time 0). Clots were observed visually, and clot disintegration was scored after 30 and 60 min on a scale from 0 (no clot), to 1 (partly disintegrated clot) and 2 (intact clot). Clot disintegration between the strains are statistically significantly different at time point 60 min (*p* = 0.0247). Data from eight experiments are shown (median with interquartile range) and were evaluated using an unpaired *t* test. *B*, stationary phase *S. pyogenes* AP1 bacteria and SIC^−^ bacteria (2 × 10^9^ cfu/ml in 13 mm citrate buffer) were incubated in plasma at 37 °C, 5% CO_2_. At various time points 10 μl of the incubations was withdrawn, diluted in PBS, and plated on TH-agar. Plates were incubated overnight at 37 °C, 5% CO_2_, and cfu were counted. Data shown are mean values ± S.D. of three experiments. Data were evaluated using an unpaired *t* test.

To analyze the effect of SIC on clotting and bacterial survival within clots, *S. pyogenes* of the AP1 strain (expressing SIC) and an AP1 SIC mutant strain (SIC^−^) (31) were used. The two strains were incubated with human plasma for 30 min at 37 °C followed by the addition of human thrombin to induce clot formation. Clots were washed, incubated in Todd Hewitt (TH) growth medium at 37 °C, and fibrinolysis of the clots was then followed over time. In clots containing entrapped mutant SIC^−^ bacteria, clot disintegration was visible after 30 min, whereas the clots with WT AP1 bacteria were still almost intact also after 60 min (Fig. 6*A*). At this time point very little remained of the clots with SIC^−^ bacteria (Fig. 6*A*). These findings are in accordance with the result given above, where SIC was shown to block streptokinase activation of plasminogen (see Fig. 4, *A* and *B*). Moreover, in the TH broth surrounding the clots with entrapped AP1 bacteria, there was a significantly higher number of bacteria recovered after 60 min as compared with the SIC^−^ strain (Fig. 6*C*). Because there were similar numbers of bacterial cells at the start of the experiment (Fig. 6*B*), the results suggest that WT bacteria were more viable than SIC^−^ mutant bacteria when released into the medium through fibrinolysis. In parallel, the number of bacterial cells within plasma clots was also determined. Here, clots were formed and incubated in TH broth as described above. At different time points the clots were washed and the fibrin network was resolved by trypsin digestion. There was no difference in the number of bacterial cells within the clots at time point 0 min (Fig. 6*D*). However, a significant higher number of AP1 bacteria remained in the clots after 60 min of incubation in TH growth medium as compared with clots with SIC^−^ bacteria (Fig. 6*D*). In addition, visual scoring showed a significant difference in the integrity of the clots between the strains after 60 min (Fig. 7*A*), a result in line with the data of Fig. 6. The growth of AP1 and SIC^−^ bacteria was also followed in plasma, without the addition of thrombin (Fig. 7*B*). During the first hours of growth, there was no difference in bacterial multiplication, whereas after 3 h a significantly higher multiplication rate was observed for the AP1 strain (Fig. 7*B*).

**Figure 8.**
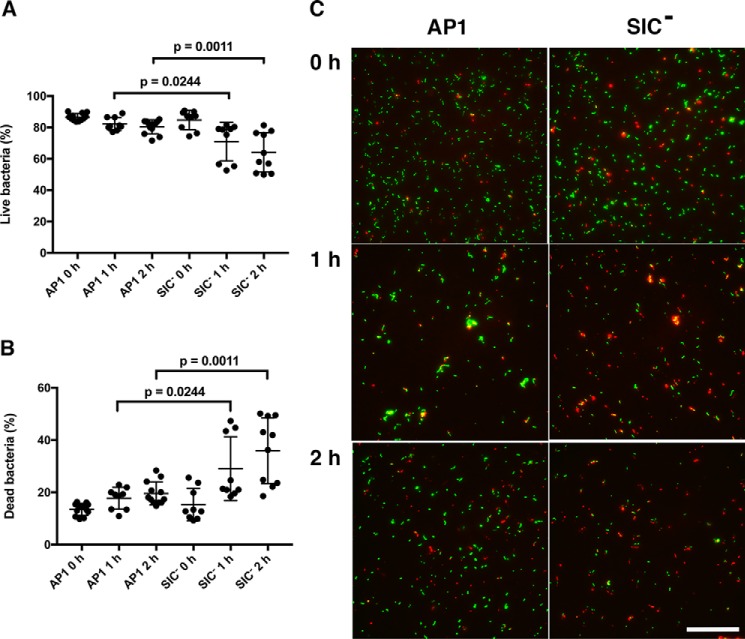
**SIC enhances survival of *S. pyogenes* within clots.** Plasma clots entrapping *S. pyogenes* bacteria were prepared for fluorescence microscopy. At the indicated time points, clots were washed, the fibrin network was resolved by trypsin treatment, and the Live/Dead staining dyes SYTO9 and propidium iodide were added to the bacterial suspensions. *A*, number of live bacteria. *B*, number of dead bacteria. Data from three experiments, at time points 0 h and 1 h, and from two experiments, at time point 2 h, are shown (median with interquartile range) and were evaluated using the Mann–Whitney test. To determine the number of live and dead bacteria in the clots, at least four images were recorded for each time point. *C*, representative fluorescence micrographs from each time point are shown. *Scale bar* represents 50 μm.

The viability of bacteria within fibrin clots was also investigated using Live/Dead staining and fluorescence microscopy. Clots were formed as described above. At different time points they were washed and treated with trypsin, and bacterial survival within the clots was determined (Fig. 8, *A* and *B*). The bacteria of the SIC^−^ strain were rapidly killed within the clots as compared with the WT SIC-expressing AP1 strain. After 1 h, 29% of the SIC^−^ bacteria were dead, and after 2 h 36% of the SIC^−^ bacterial cells were nonviable (Fig. 8*B*). In comparison, the AP1 strain survived longer within the clots, and after 2 h only 20% of these bacterial cells were defined as dead (Fig. 8*B*). Representative fluorescence micrographs of AP1 and SIC^−^ bacteria at each time point are shown in Fig. 8*C*. Taken together, these data demonstrate that SIC through interaction with plasminogen inhibits fibrinolysis, which prolongs the survival of bacteria within fibrin clots.

### SIC and systemic S. pyogenes infection in mice

**Figure 9.**
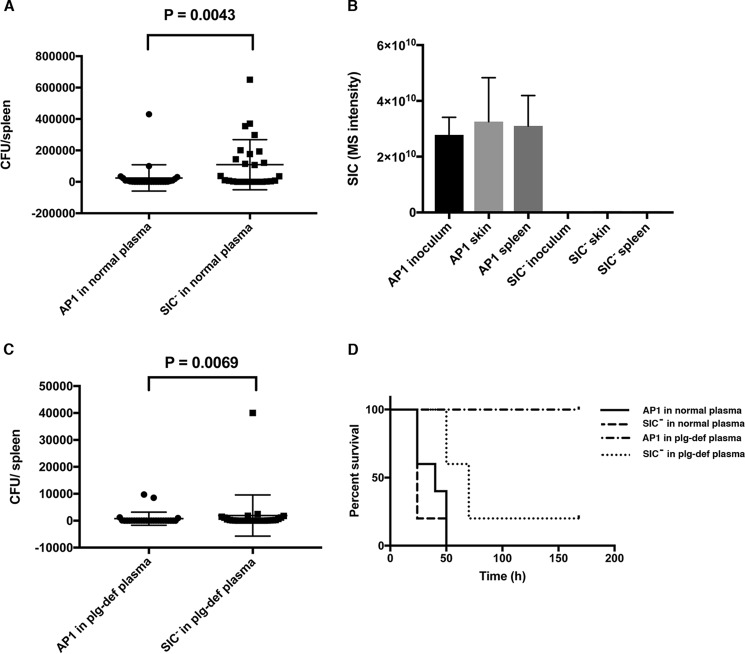
**SIC and systemic *S. pyogenes* infection in mice.** Female C57/BL6J mice were injected subcutaneously with mid-logarithmic *S. pyogenes* strains AP1 and SIC^−^ (2.5 × 10^4^ cfu/animal) in 250 μl of human plasma or human plasminogen-deficient plasma. The animals were sacrificed after 24 h, and the total number of cfu in the spleens was determined. Alternatively, the survival of mice was followed for 7 days. *A*, the number of cfu in the spleens of mice infected with the SIC^−^ strain in human plasma was significantly higher compared with mice infected with the AP1 strain (*p* = 0.0043). *B*, bacterial supernatants from *S. pyogenes* inoculum strains and mouse-passaged derivative strains isolated from either spleen or skin were analyzed with shotgun proteomics in three replicates for AP1 and one replicate for SIC^−^. The MS intensity of protein SIC is determined using the MaxQuant computational platform for MS-based shotgun proteomics. The plot shows SIC protein intensity ± S.D. *C*, the number of cfu in spleens of mice infected with the SIC^−^ strain in plasminogen-depleted plasma was significantly higher compared with mice infected with the AP1 strain (*p* = 0.0069). For both strains the number of cfu in spleens from mice infected in the presence of plasminogen-depleted plasma is significantly lower as compared with mice infected in the presence of normal plasma (AP1, *p* < 0.0001; SIC^−^, *p* < 0.0001). Data from six individual experiments (total number of mice/group = 27) are shown (median with interquartile range); the data were evaluated using the Mann–Whitney test. *D*, survival of infected mice (5 animals/group) was followed for 7 days.

To examine the role played by SIC upon fibrinolysis in a more complex *in vivo* setting, we used a mouse model of subcutaneous infection, where AP1 or the SIC^−^ bacteria were administrated together with human plasma or plasminogen-deficient plasma, respectively. The rationale for using this model is that streptokinase is highly specific for human plasminogen and does not activate mouse plasminogen (44, 45). Such a model has also been used previously to demonstrate the crucial role of plasminogen and streptokinase in the pathogenesis of *S. pyogenes* (44, 46). Mice were infected subcutaneously with 2.5 × 10^4^ bacteria, grown to mid-log phase, together with plasma. The animals were sacrificed 24 h post-infection, spleens were recovered, and the number of colony forming units (cfu) within the spleens was determined. In the presence of normal human plasma, significantly more bacterial cells were recovered from the spleens of mice infected with the mutant SIC^−^ strain as compared with the WT SIC-expressing AP1 strain (Fig. 9*A*). This result suggests a more rapid systemic spread of SIC^−^ bacteria, most likely as a result of fibrinolysis. As expected, Western blot analysis of SIC expression by bacteria isolated from spleen showed secretion of SIC by AP1 but not by SIC^−^ mutant bacteria (data not shown). The secretion of SIC by bacteria from the inoculum, the local subcutaneous site of infection and spleen, was quantified by MS. Although there was a tendency toward increased SIC production at the local site as compared with the inoculum and the spleen, this increase was not statistically significant. Again, mutant bacteria showed no secretion of SIC (Fig. 9*B*). When the bacterial strains were administered together with plasminogen-deficient plasma, the bacterial spread from the local site was significantly lower for both bacteria. However, compared with the AP1 strain, a significantly higher number of cfu was again detected in the spleens of animals infected with the mutant SIC^−^ strain (Fig. 9*C*).

**Figure 10.**
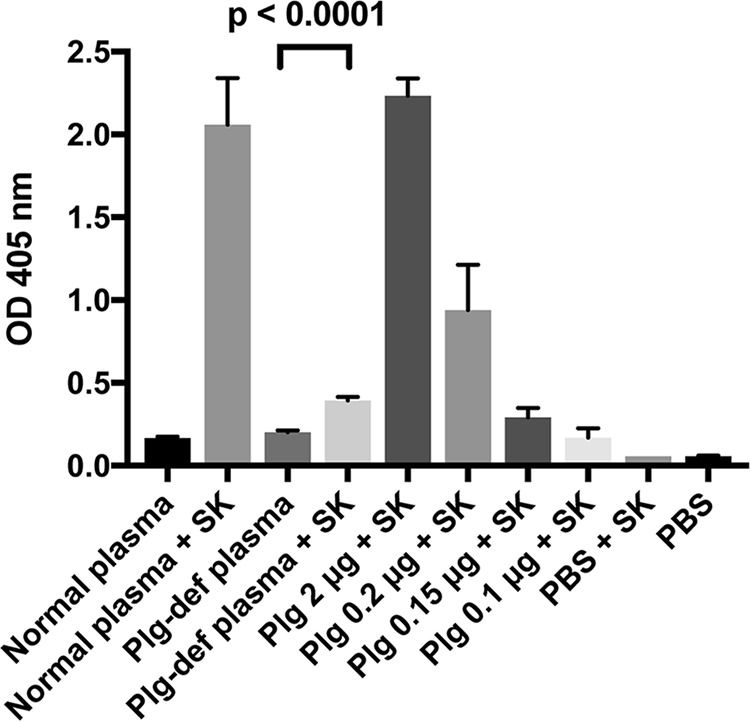
**Streptokinase activation of plasminogen in plasma.** Normal plasma (20 μl), plasminogen-deficient plasma (20 μl), various amounts of plasminogen, and PBS were incubated in microtiter wells with streptokinase (8 units) for 30 min at 37 °C. As background control, the plasma was incubated in PBS only. Chromogenic substrate S-2251, specific for activated plasminogen, was added, and incubation continued for another hour at 37 °C. Cleavage of the substrate was analyzed by measuring the absorbance at 405 nm. Data shown are mean values ± S.D. of three individual experiments (samples in triplicate) and were evaluated using the Mann–Whitney test.

Next, we infected mice as described above, and the survival of the animals was recorded for 7 days. In the presence of human plasminogen all mice infected with the SIC-expressing AP1 strain were dead by 50 h post-infection, whereas mice infected with the same bacteria in the absence of plasminogen were still healthy at 7 days post-infection (Fig. 9*D*). The mutant SIC^−^ strain displayed slightly enhanced virulence in the presence of plasminogen (Fig. 9*D*) in accordance with Fig. 9*A*. Again, in the absence of human plasminogen, the SIC^−^ strain was still more virulent as compared with the AP1 strain. According to the manufacturer, the concentration of plasminogen in plasminogen-deficient plasma is <0.01 unit/ml; using a chromogenic substrate, a low level of plasminogen that could be activated by streptokinase was detected also in the depleted plasma (Fig. 10). Thus, this trace amount of activated plasminogen is sufficient to induce fibrinolysis in clots entrapping SIC^−^ bacteria and facilitate dissemination.

## Discussion

In response to bacterial infection and inflammation the coagulation system is activated, and at the site of infection fibrin clot formation leads to bacterial entrapment. In animal experiments this has been demonstrated to play an important role in preventing the dissemination of *S. pyogenes* bacteria (19). Many bacterial species including *S. pyogenes* have, however, evolved strategies to counteract clot entrapment by the binding and activation of plasminogen, whereby the dissolution of fibrin clots is promoted (37, 47, 48). The binding of plasminogen/plasmin to the surface of *S. pyogenes* bacteria can be mediated by the protein PAM (49), a member of the M protein family, or by surface-bound enzyme molecules such as glyceraldehyde-3-phosphate dehydrogenase (50, 51) and α-enolase (52). In addition, through the binding of fibrinogen to M protein, *S. pyogenes* may recruit plasminogen in complex with streptokinase to its surface (42, 43). Such recruitment of plasminogen/plasmin has been suggested to play an important role in *S. pyogenes* pathogenesis (48, 53). Once bound to or generated at the bacterial surface, plasmin will degrade the fibrin network in the encapsulating fibrin clot, thereby promoting bacterial dissemination.

In the present work we found that SIC interferes with the fibrinolytic system. Through the inhibition of plasminogen activation by streptokinase, SIC delays fibrinolysis and enhances bacterial survival within the clot. Flow cytometry analyses and fluorescence microscopy demonstrated that SIC and plasminogen co-localize at the surface of *S. pyogenes* bacteria. The complex formed between plasminogen and streptokinase involves sites in the catalytic domain and the kringle domains of plasminogen. The binding of another plasminogen molecule can be inhibited by lysine analogues such as 6-aminocaproic acid, suggesting that the kringle domains are involved (8). The binding of SIC to plasminogen can be inhibited by aminocaproic acid, indicating that the interaction takes place in the kringle domains. SIC may therefore not only interfere with complex formation between plasminogen and streptokinase but also with the binding of a second plasminogen molecule to the complex. As a result, the activation of plasminogen is reduced.

In our work SIC did not inhibit the activation of plasminogen by host tPA, the major circulating activator, suggesting that SIC does not block the activation of plasminogen in the circulation. Streptokinase is expressed by all *S. pyogenes* isolates and is specific for human plasminogen. From an evolutionary point of view, this is noteworthy, that *S. pyogenes* infects only humans. Phylogenetic studies have grouped streptokinase from different strains into two clusters, type 1 and type 2, with type 2 divided into subclusters: type 2a is associated with M1-expressing strains and type 2b with protein PAM–expressing strains (54). Whereas streptokinase expressed by cluster 1 strains can activate plasminogen in solution, fibrinogen is required for the activation of plasminogen by streptokinase type 2 (54). Thus, the reduced activation of plasminogen in the presence of SIC might also reflect a diminished recruitment of plasminogen to M1-bound fibrinogen.

The immobilization and killing of *S. pyogenes* bacteria within fibrin clots has been shown to be dependent on HRG (21). It has also been demonstrated that peptides released from fibrinogen by thrombin are antimicrobial (18). However, only fibrinogen-binding bacteria are affected, suggesting that such peptides are active within a fibrin clot (18). Kininogen-derived antibacterial peptides, generated via contact activation at bacterial surfaces, also play a role in bacterial killing (55). Our present data demonstrate that the SIC^−^ strain is rapidly killed within the fibrin clot as compared with SIC-expressing bacteria. In this context, it is interesting to note that SIC inhibit the bactericidal activity of both HRG and kininogen-derived peptides (21, 32), suggesting that this property of SIC contributes to the survival of AP1 bacteria within the clots. Possibly, SIC also blocks the activity of fibrinogen-derived peptides. Within the clot, the secreted SIC binds to fibrin fibers via its central region and to plasminogen via its C-terminal part, leaving the N-terminal region of SIC free to bind and block the action of antibacterial proteins/peptides that are present or generated upon clot formation.

The M1 serotype is more frequently associated with severe invasive infections and sepsis caused by *S. pyogenes.* In these conditions the activation of coagulation will lead to recruitment and activation of neutrophils and platelets, resulting in thrombus formation (56, 57). SIC is produced during the early growth phase of *S. pyogenes* bacteria of the M1 serotype (27), and the *sic* gene is also up-regulated when the bacteria are exposed to saliva or blood (58, 59). Within encapsulating fibrin clots, multiplying SIC-expressing *S. pyogenes* will be protected from the action of antibacterial peptides. The delayed fibrinolysis promotes bacterial enrichment within the clot, and once fibrinolysis is initiated a larger number of living bacteria will spread to more favorable growth sites. On the other hand, strains lacking SIC are killed to a higher degree within fibrin clots.

Previous animal experiments have demonstrated that the SIC^−^ strain is less virulent in mice as compared with the WT AP1 strain (31). In the present study a different model was required to examine the role of SIC modulation of fibrinolysis *in vivo*. Because streptokinase is specific for human plasminogen, the bacteria were administrated together with human plasma. The results obtained with the SIC-expressing AP1 strain are in accordance with previously reported data on the significant effect of plasminogen on *S. pyogenes* virulence (44, 46, 53). Moreover, the slightly enhanced systemic spread of SIC^−^ bacteria in the presence of plasminogen supports the *in vitro* results showing that fibrinolysis is promoted in absence of SIC. Surprisingly, also in plasminogen-deficient plasma the SIC^−^ strain was more virulent than the AP1 strain (Fig. 9). However, in the depleted plasma, trace amounts of plasminogen are present and can be activated by streptokinase, and these trace amounts of plasminogen are apparently sufficient for SIC^−^ bacteria to induce fibrinolysis and promote systemic spread in the animals. In contrast, the AP1 strain secretes SIC, which efficiently binds the low concentration of plasminogen and thereby blocks its activation. Moreover, at the local infection site, inflammation leads to the recruitment of neutrophils and induces an exudation of murine plasma. Generally, murine coagulation factors are very similar to their human counterparts (60), suggesting that activation of the murine coagulation system may also contribute to entrapment of *S. pyogenes* over time. Presumably, the activation of murine plasminogen would result in fibrinolysis and the spread of SIC^−^ bacteria. Furthermore, human and murine plasminogen display 79% homology (60), indicating that SIC interferes also with the activation of murine plasminogen.

To summarize, the targeting of coagulation and fibrinolysis demonstrated here identifies novel properties of SIC, suggesting that SIC plays its major role at an early stage of infection and promotes the persistence of bacteria at the site of infection. Our data lend further support to the notion that the activity of this multifaceted protein in part may explain the high frequency of *S. pyogenes* strains of the M1 serotype, both in general and in severe invasive infections.

## Materials and methods

### Bacteria and growth conditions

The *S. pyogenes* strain AP1 (40/58) of serotype M1 was obtained from the World Health Organization Collaborating Centre for Reference and Research on Streptococci, Prague, Czech Republic. The mutant strain SIC^−^ has been described previously (31). Bacteria were cultivated in TH broth (Difco) at 37 °C, 5% CO_2_. For the cultivation of the mutant SIC^−^ strain, kanamycin was added to a final concentration of 150 μg/ml.

### Proteins, antibodies, reagents, and iodination

Protein SIC was purified from the growth medium of the AP1 strain as described previously (27). Recombinant AP1-SIC and recombinant SIC fragments I, II, and III were prepared by introducing the DNA sequences corresponding to residues Glu^1^–Thr^273^ (intact SIC), Glu^1^–Thr^73^ (fragment I), Glu^70^–Gln^166^ (fragment II), and Ser^167^–Thr^273^ (fragment III), respectively, into the expression vector pNIC-Bsa4 using ligation-independent cloning (61). The resulting expression constructs contained a His tag capture sequence and a tobacco etch virus (TEV) protease cleavage site at the N terminus preceding the expressed protein of interest. *E. coli* cells were cultured in Terrific Broth medium with 50 μg/ml kanamycin. Protein expression was induced with 0.5 mm isopropyl-β-thiogalactopyranoside, and cell growth was continued for 18 h at 18 °C. Harvested bacterial pellets were suspended in lysis buffer (100 mm Tris, pH 8.0, 300 mm NaCl, 10 mm imidazole, and 0.5 mm TCEP) complemented with EDTA-free protease inhibitor and ruptured using a French press cell disruptor. Cell debris and membrane components were removed by centrifugation at 40,000 × *g* for 30 min. Purification of the proteins was performed in two steps, with the first step employing immobilized metal ion affinity chromatography on an ÄKTA express system (GE Healthcare) at 4 °C with HiTrap chelating columns (62). The purified intact SIC and the SIC fragments were subjected thereafter to treatment with TEV protease to remove the His tag. The final purifications were performed by reverse phase chromatography on a Dionex Ultimate 3000 HPLC system equipped with a preparative C18 HPLC column (Gemini NX, Phenomenex). The proteins were eluted by a linear gradient of 10–60% acetonitrile, flash-frozen, and lyophilized. The purity and monodispersity of the recombinant proteins were confirmed by SDS-PAGE and MS. The kininogen-derived peptide NAT26 has been described previously (41). Human prothrombin and thrombin were from Innovative Research. HSA and human fibrinogen were from Sigma. Human Glu-Plasminogen, plasmin, normal plasma, and plasminogen-deficient plasma were from Hemochrom Diagnostica, and streptokinase was purchased from Sigma. Proteins were radiolabeled with ^125^I using the IODO-BEAD® iodination reagent (Pierce) as described by the manufacturer.

### Slot binding

Proteins or peptides were applied directly to PVDF membranes using a MilliBlot-D system (Millipore). Membranes were blocked in TBS (0.05 m Tris-HCl, pH 7.5, and 0.15 m NaCl) containing 3% BSA (Sigma), incubated with ^125^I-labeled protein (2 × 10^5^ cpm/ml in TBS + 3% BSA) for 3 h at room temperature or at 4 °C overnight. Membranes were then washed with TBS containing 0.05% Tween 20, and binding was visualized using the Fuji FLA-3000 imaging system.

### Clotting assay and chromogenic substrate assay

Fibrinogen polymerization was measured in a coagulometer (Amelung). A thrombin clotting time reagent (Hemochrom Diagnostica) was preincubated with various amounts of SIC (up to 10 μg) or 20 mm Tris-HCl buffer, pH 7.5, in a total volume of 100 μl for 30 min at 37 °C. The mixture was then added to 100 μl of citrated plasma or 100 μl of fibrinogen solution (3 mg/ml in 13 mm citrate buffer), and clotting time was measured.

For analyses of thrombin activity, 100 μl of fibrinogen solution (3 mg/ml in 13 mm citrate buffer) was incubated with 60 μl of thrombin clotting time reagent and 40 μl of SIC solution (various amounts of SIC up to 10 μg) or buffer for 15 min at 37 °C. The samples were centrifuged for 5 min at 13,000 × *g*, and supernatants were removed. Clots were resuspended in 100 μl of S-2238, a chromogenic substrate for thrombin, and incubated for 30 min at room temperature. Following centrifugation for 5 min at 13,000 × *g*, the supernatants were transferred to wells in microtiter plates (Nunc MaxiSorp), and absorbance at 405 nm was measured.

Plasminogen (2 μg) was incubated with streptokinase (8 units) and various amounts of SIC or 20 mm Tris-HCl buffer, pH 7.5, in a total volume of 20 μl for 1 h at 37 °C. One volume of 100 μl of S-2251, a chromogenic substrate for streptokinase-activated plasminogen, was added, and incubation continued for 1 h at 37 °C. From each sample 100 μl was transferred to wells in microtiter plates, and the absorbance at 405 nm was measured. For analysis of plasminogen activation in human plasma, 20 μl of normal plasma, 20 μl of plasminogen-deficient plasma, and various concentrations of purified plasminogen or buffer in a total volume of 80 μl were incubated with 8 units of streptokinase in microtiter well plates for 30 min at 37 °C. Twenty μl of S-2251 was added, and incubation continued for 1 h at 37 °C. The absorbance at 405 nm was measured.

### ELISA

Microtiter plates were coated with 200 μl of human plasminogen (0.6 μg/ml) overnight at 4 °C. The plates were washed with PBST (PBS supplemented with 0.05% Tween 20) and then blocked with blocking buffer (PBST + 2% BSA) for 30 min at room temperature. Then the plates were incubated with 100 μl of SIC (2 μg/ml in blocking buffer) in the presence of 100 μl of various inhibitors diluted in blocking buffer. Bound SIC was detected with polyclonal rabbit antisera against SIC (1:8000 dilution in blocking buffer) followed by horseradish peroxidase–conjugated secondary antibodies against rabbit IgG (1:3000 dilution in blocking buffer). All incubations were performed for 1 h at 37 °C followed by a washing step. The substrate solution was added, and following incubation for 30 min at room temperature the absorbance at 415 nm was measured.

### Binding of SIC to fibrin clots

One hundred μl of fibrinogen solution (3 mg/ml in 20 mm Tris-HCl, pH 7.5) or 100 μl of citrate-treated plasma was incubated with human thrombin (0.2 unit) in plastic tubes (Sarstedt) at 37 °C for 30 min until clots were formed. The clots were washed three times with 20 mm Tris-HCl, pH 7.5, and then incubated with 125 μl of ^125^I-labeled SIC (∼10,000 cpm) in PBST for 30 min at room temperature. Two ml of PBST was added, the clots were collected by centrifugation at 1600 × *g* for 10 min at room temperature, and bound radioactivity was measured in a gamma counter.

### Fibrinolysis

Clots were formed by incubating 50 μl of fibrinogen solution (3 mg/ml in 20 mm Tris-HCl, pH 7.5) with 50 μl of human thrombin solution (containing 0.1 unit of thrombin) in microwell plates at 37 °C for 1.5 h. The absorbance at 405 nm was measured (corresponding to time point zero) and then plasminogen (0.5 μg) and streptokinase (8 units) were added together with various concentrations of SIC in a volume of 100 μl, and incubation at 37 °C was continued for another 3 h. The absorbance at 405 nm was measured and related to the initial value at time point zero.

### Bacterial entrapment and fibrinolysis of clots

Stationary phase bacteria of the AP1 strain and the mutant SIC^−^ strain were washed and resuspended in 13 mm sodium citrate buffer to a concentration of 2 × 10^9^ cfu/ml. A volume of 500 μl was incubated with 500 μl of citrate-treated plasma for 30 min at 37 °C, 5% CO_2_ under rotation. Human thrombin (0.8 unit) was added, and incubation continued for another 30 min at 37 °C until a clot was formed. The clot was washed twice with 13 mm sodium citrate buffer followed by the addition of 1000 μl of TH broth. At various time points, the samples were examined visually and photographed. At the starting time and after 1 h, 10 μl withdrawn from the TH broth surrounding the clot was diluted in PBS and plated on TH-agar. Plates were incubated overnight at 37 °C, 5% CO_2_, and cfu were counted.

For quantification of the bacterial cells within clots, clots were formed as above with 200 μl of bacterial solution and 200 μl of citrate-treated plasma followed by the addition of human thrombin. After a washing step, the clots were incubated in 200 μl of TH broth. At various time points, the clots were washed with 13 mm sodium citrate buffer and incubated at 37 °C with 100 μl of trypsin (4 mg/ml) for 15 min until the fibrin network was resolved. One hundred μl of trypsin inhibitor (4 mg/ml) was added, and serial dilutions of the suspensions were plated onto TH-agar plates, which were incubated at 37 °C, 5% CO_2_ overnight. The number of cfu was counted.

### Bacterial growth in human plasma

The stationary phase bacteria of the AP1 strain and the mutant SIC^−^ strain were washed with 13 mm citrate buffer and resuspended to a concentration of 2 × 10^9^ cfu/ml. A volume of 500 μl was incubated with 500 μl of citrate-treated plasma at 37 °C, 5% CO_2_ under rotation. At various time points, 10 μl was withdrawn, diluted in PBS, and plated on TH-agar. Plates were incubated overnight at 37 °C, 5% CO_2_, and cfu were counted.

### Flow cytometry

Human plasminogen and protein SIC were labeled with Alexa Fluor 488 and Alexa Fluor 633 labeling kits (Molecular Probes), respectively, according to the manufacturer's instructions. Stationary phase bacteria of the AP1 strain were washed twice with PBS and resuspended to a concentration of 2 × 10^7^ cfu/ml. One volume of 100 μl was incubated with labeled plasminogen and labeled SIC for 30 min in the dark at room temperature. Following incubation 400 μl of PBS was added, and samples were analyzed on a BD Accuri flow cytometer using logarithmic acquisition. Alternatively, samples were centrifuged at 1000 × *g* for 10 min, resuspended in 500 μl of PBS, and analyzed. For each sample 30,000 events were recorded, and the results were analyzed using Accuri C6 software (BD Biosciences).

### Fluorescence microscopy

Bacterial samples prepared for flow cytometry (see above) were fixed with paraformaldehyde (4% final concentration) for 45 min at room temperature. The samples were washed with blocking buffer (PBS + 5% BSA) and resuspended in 50 μl of the same buffer. Samples were absorbed onto poly-l-lysine–precoated coverslips for 1 h at room temperature and then mounted using ProLong Gold Antifade Mountant with 4′,6-diamidino-2-phenylindole (DAPI, Thermo Fisher Scientific). Samples were analyzed using a Nikon Eclipse Ti2 fluorescence microscope with a Plan Apo λ 100× oil/1.45 NA objective and Andor Zyla camera.

Bacterial viability within the clots was quantified by the use of a Live/Dead® BacLight^TM^ bacterial viability kit (Molecular Probes). Clots were prepared as described above; following clot dissolution equal volumes (2 μl) of the dyes SYTO9 and propidium iodide were added to the bacterial suspensions (200 μl). After incubation for 15 min in the dark at room temperature, 5 μl of the sample was added onto a slide, covered with a coverslip, and observed in the fluorescence microscope using a Plan Apo λ 40×/0.95 NA objective. Analysis was performed using Imgel software.

### Animal experiments

WT C57/BL6J female mice were purchased from Scanbur and housed in the animal facility at Lund University. Experiments were carried out when the mice were 9 weeks old. Procedures were approved by the local ethics committee (Lund University, Lund, Sweden). *S. pyogenes* strains AP1 and SIC^−^ strain were grown overnight in TH broth at 37 °C, 5% CO_2_. An aliquot of these cells was added to fresh TH broth and cultivated until *A*_620_ = 0.4. The bacterial cells were washed twice with PBS, resuspended to a concentration of 2 × 10^9^ cfu/ml, and then further diluted to 0.5 × 10^7^ cfu/ml. From these bacterial solutions 20 μl was added to 980 μl of normal plasma or plasminogen-deficient plasma, respectively, from which 250 μl was immediately injected subcutaneously into each mouse (2.5 × 10^4^ cfu). The bacterial dose was confirmed by the plating of the inoculum for each individual experiment. To determine bacterial dissemination from the local site of infection, animals were sacrificed 24 h post-infection. The spleen was removed and homogenized in 0.5 ml of PBS using Magnalyzer (Roche). Another 0.5 ml of PBS was added, and the suspension was further diluted with PBS. The bacterial load was quantified by plating serial dilutions of incubation mixtures on blood agar, incubating the plates at 37 °C, 5% CO_2_ overnight, and counting the cfu. For survival studies the animals were monitored closely on a daily basis for signs of infection and sacrificed at near death (weight loss of 15–20% and behavioral changes).

Bacteria isolated from the inoculum, the subcutaneous local site, and the spleen were grown overnight in TH broth at 37 °C, 5% CO_2_. An aliquot of these cells was added to fresh TH broth and cultivated until *A*_620_ = 0.4. The supernatants were collected by centrifugation, sterile-filtered, and analyzed by MS for the presence of protein SIC (see below). Supernatants were also analyzed by Western blotting using antibodies against protein SIC.

### Mass spectrometry analysis

The bacterial supernatants were mixed with SDS (final concentration of 1%) and incubated at 80 °C for 10 min for complete dissolution and then concentrated using an Amicon Ultra centrifugal filter with a 30-kDa cutoff (Millipore). The concentrated protein samples were trypsin-digested using the filter-aided sample preparation (FASP) method (63). The resulting peptides were desalted and cleaned with reversed-phase spin columns (Solaμ HRP, Thermo Fisher Scientific) followed by analysis with a Q Exactive Plus mass spectrometer (Thermo Fisher Scientific) connected to an EASY-nLC 1000 Ultra-HPLC system (Thermo Fisher Scientific). Peptides were separated on EASY-Spray ES802 columns (Thermo Fisher Scientific) using a linear gradient from 3 to 35% acetonitrile in aqueous 0.1% formic acid during 1 h. Data-dependent acquisition instrument settings were identical to those described elsewhere (64). Raw MS files were processed with MaxQuant v1.5.2.8 and searched against the *S. pyogenes* AP1 protein database from GenBank^TM^ accession No. CP007537.1 with PATRIC (https://www.patricbrc.org)^4^ as the annotation source (65). The false discovery rates for both the peptides and the proteins were set at 0.01.

### Statistical analysis

GraphPad Prism 6 was used to perform statistical analysis, and significance was tested using the Mann–Whitney test and an unpaired *t* test.

## Author contributions

I.-M. F. conceived and designed the study, performed and analyzed experiments, wrote the manuscript; O. S. performed animal experiments, reviewed the results and approved the final version of the manuscript; A. N. performed fluorescent microscopy analyses, reviewed the results and approved the final version of the manuscript; C. K. performed and analyzed Mass spectrometry experiment, reviewed the results and approved the manuscript; M. W. purified protein SIC fragments, reviewed results and approved the manuscript; L. B. conceived and designed the study, contributed in writing the manuscript.

## Footnotes

4 Please note that the JBC is not responsible for the long-term archiving and maintenance of this site or any other third party–hosted site.

## Footnotes

3 The abbreviations used are:

tPA: tissue-type plasminogen activator
cfu: colony forming unit(s)
HK: high molecular weight kininogen
HRG: histidine-rich glycoprotein
HSA: human serum albumin
TEV: tobacco etch virus
TH: Todd Hewitt
PVDF: polyvinylidene difluoride
TCEP: tris(2-carboxyethyl)phosphine.
